# A European inter-laboratory trial to evaluate the performance of three serological methods for diagnosis of *Mycoplasma bovis* infection in cattle using latent class analysis

**DOI:** 10.1186/s12917-019-2117-0

**Published:** 2019-10-25

**Authors:** Anna-Maria Andersson, Anna Aspán, Henk J. Wisselink, Bregtje Smid, Anne Ridley, Sinikka Pelkonen, Tiina Autio, Klara Tølbøll Lauritsen, Jane Kensø, Patrice Gaurivaud, Florence Tardy

**Affiliations:** 1 National Veterinary Institute (SVA), Uppsala, Sweden; 2 Wageningen Bioveterinary Research, P.O. Box 65, 8200 AB Lelystad, the Netherlands; 3 Animal and Plant Health Agency (APHA), Surrey, UK; 4 Finnish Food Authority, Kuopio, Finland; 5 National Veterinary Institute, Technical University of Denmark, Kgs Lyngby, Denmark; 6 Université de Lyon, Anses, Laboratoire de Lyon, UMR Mycoplasmoses des ruminants, Lyon, France

**Keywords:** *Mycoplasma bovis* cattle, Inter-laboratory trial, ELISA, Western blot, Latent class analysis

## Abstract

**Background:**

*Mycoplasma bovis* (*M. bovis*) is an emerging bovine pathogen, leading to significant economic losses in the livestock industry worldwide. Infection can result in a variety of clinical signs, such as arthritis, pneumonia, mastitis and keratoconjunctivitis, none of which are *M. bovis*-specific. Laboratory diagnosis is therefore important. Serological tests to detect *M. bovis* antibodies is considered an effective indicator of infection in a herd and often used as a herd test. Combined with clinical judgement, it can also be used to implement control strategies and/or to estimate the disease prevalence within a country. However, due to lack of harmonisation of approaches to testing, and serological tests used by different laboratories, comparisons of prevalence data between countries is often difficult. A network of researchers from six European countries designed and participated in an inter-laboratory trial, with the aim of evaluating the sensitivity (*Se*) and specificity *(Sp)* of two commercially available ELISA tests (ID Screen® ELISA (IDvet) and BIO K302 ELISA (BIO-X Diagnostics)) for diagnosis of *M. bovis* infection. Each laboratory received a blinded panel of bovine sera and tested independently, according to manufacturer’s instructions. Western blot analyses (WB) performed by one of the participating laboratories was used as a third diagnostic test in the statistical evaluation of *Se* and *Sp* values using latent class analysis.

**Results:**

The *Se* of WB, the ID Screen® ELISA and the BIO K302 ELISA were determined to be 91.8, 93.5 and 49.1% respectively, and corresponding *Sp* of the three tests were 99.6, 98.6 and 89.6%, respectively.

**Conclusions:**

The present study is, to our knowledge, the first to present an inter-laboratory comparison of the BIO K302 ELISA and the ID Screen® ELISA. Based on our results, the ID Screen® ELISA showed high consistency with WB and performed with higher precision and accuracy than the BIO K302 ELISA.

## Background

*Mycoplasma bovis* has emerged as a pathogen of increasing importance in many industrialised countries around the world, causing significant economic and production losses particularly in the beef and dairy industries [1–4]. Infection with *M. bovis* is associated with a variety of clinical manifestations. In calves, the infection can present as respiratory disease, arthritis and otitis media. In adult cattle, pneumonia, mastitis, otitis media, and reproductive problems have been observed [1, 4, 5]. Since none of these clinical signs are pathognomonic, definitive and accurate diagnosis requires laboratory confirmation. This is important for implementation of control strategies such as enabling immediate separation of infected livestock and early administration of appropriate treatment, as the spread of disease is difficult to contain once present on a farm [6, 7].

Bacterial cultural identification has traditionally been considered the gold standard method for *M. bovis,* but is labour intensive and time-consuming [8]. Interpretation of culture results can also be hampered by the intermittent shedding of *M. bovis*, sub-optimal sampling and transportation procedures or by antimicrobial treatment prior to sampling [1, 7]. PCR-based detection of *M. bovis* has been increasingly favoured over the past two decades to overcome difficulties associated with cultural diagnosis. However, PCR methods are also highly dependent on the organism being shed at the time of sampling, as well as efficiency of DNA extraction, particularly in presence of inhibitors, as well as specific primers and probes with sensitive detection [7, 9].

With demand for rapid, inexpensive and convenient tests, serological tests for herd level testing have been developed, and used widely, over several decades. These methods are designed to retrospectively detect *M. bovis* antibodies in cattle that have been exposed to the pathogen and thus have mounted a detectable humoral immunological response (usually from 2 to 3 weeks after infection) [7]. The immunological response is theoretically measurable in plasma, serum or milk although effectiveness of detection may vary depending on sample type and format of the test [7]. Used alone, these serological tests enable rapid and cost-effective screening for the presence of infection or demonstration of absence of infection in a herd. However, for optimal monitoring of *M. bovis* infection status in a herd, a combination with other diagnostic methods is recommended [6].

Several serological diagnostic tests exist, each having their benefits and limitations. Western blot analysis (WB; also known as immunoblotting) has been considered a robust and specific method, suitable as a confirmatory test [10, 11], but it requires preparation of a suspension of antigenic proteins from an appropriate control strain that is then electrophoresed and blotted onto membranes before being ready to test serum samples. Therefore, the method is time consuming and not suitable for screening of large numbers of samples. For the routine laboratory workflow, use of enzyme-linked immunosorbent assays (ELISA) is often the preferred method.

The choice of the antigen(s) used in the ELISA assays is important as it must be (i) both specific for, and universally present in, all strains of the targeted bacterium, (ii) persistently expressed during the infection, and (iii) recognised by the host humoral response independently of the clinical outcome of the infection [10–12]. Antigenic variation in *M. bovis* is well recognised and many of the originally developed assays comprise whole cell antigen [13]. In the past decade, ELISA tests based on antigens that are expressed in *Escherichia coli* by recombinant DNA technology have been developed [12, 14–17]. However, in the absence of commercially standardised production methods and controls, variability associated with reproducibility of antigen coating when in-house assays are transferred to other laboratories can make comparisons problematic [8, 9]. Owing to pre-validated performance and general ease of use, commercially produced *M. bovis* ELISAs are therefore attractive and are increasingly used by diagnostic laboratories globally.

Although several *M. bovis* serological studies have been conducted, many studies report results of development of in-house assays [14, 16, 18, 19], with fewer focused on comparison of methods [10, 12], and to our knowledge none has focused on the inter-laboratory performance of these assays. Two commercially produced ELISA kits that are used internationally for the detection of *M. bovis* antibodies in cattle have featured predominantly in previously reported studies; BIO K302 (Bio-X Diagnostics, Rochefort, Belgium) and Bovicheck (Biovet Inc., Quebec, Canada), with the latter not currently used by veterinary laboratories in Europe [12, 16, 17, 20–22]. Another commercial ELISA for *M. bovis* which also uses plates coated with a purified *M. bovis* recombinant antigen has recently become available; the ID Screen® ELISA (IDvet, Grabels, France). This ELISA has, to our knowledge, not previously been evaluated by diagnostic laboratories.

The aim of the study was to evaluate the sensitivity (*Se*) and specificity *(Sp)* of two commercial ELISA kits (ID Screen® ELISA from IDvet and BIO K302 ELISA from BIO-X Diagnostics) for serodiagnosis of *M. bovis* in cattle by means of an inter-laboratory comparison. WB was used as a third method to enable statistical evaluation using latent class analysis (LCA).

## Results

### Number of positive and negative serum samples

#### Western blot analysis

**Fig. 1:**
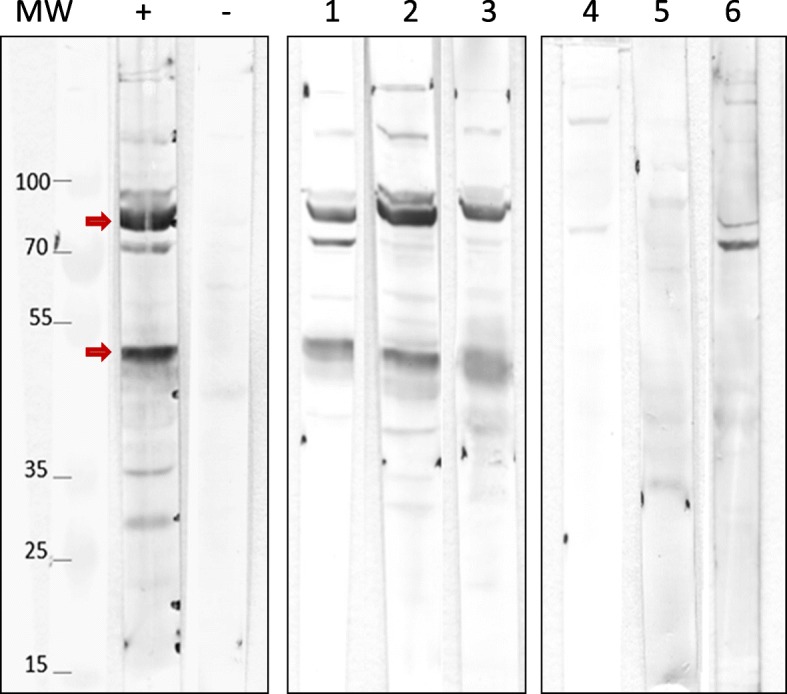
Western blot analysis (WB) of the reactivity of different sera with *M. bovis* strain L15762. MW, molecular weight (kDa); “+” and “-“are positive and negative controls, respectively. Illustration of the banding patterns obtained for positive (lanes 1 to 3) and negative sera (lanes 4 to 6). The arrows on the left point toward the two main bands expected to be present in all positive sera

Of the 180 serum samples, analysed by WB, 77 (43%) exhibited a banding pattern consistent with *M. bovis* infection and were categorised as positive, while 103 (57%) were negative, by virtue of the absence of the 50 and 85 kDa indicative immunogenic bands. This included all 90 serum samples from northern Sweden. The positive serum samples all originated from the high-prevalence area (Fig. 1; Table 1).

**Table 1:** Results of the western blot analysis (WB) as performed by laboratory 3 on the 180 serum samples. Serum samples originated from cattle populations where *M. bovis* is known to be prevalent (the high-prevalence area: Finland, France, the Netherlands and the United Kingdom) and from a cattle population which was considered highly unlikely to have been exposed to *M. bovis* (the low-prevalence area: northern Sweden)

	Number (%) of tests		
WB result	High prevalence area	Low prevalence area	All samples
Positive	77 (85.5%)	0 (0%)	77 (42.8%)
Negative	13 (14.5%)	90 (100%)	103 (57.2%)
All	90	90	180

#### ELISA I: ID screen® ELISA

Forty-four percent of the serum samples had an S/P % of ≥60% and were categorised as seropositive using the cut-off recommended by the manufacturer. The proportion of positive test results varied between 43 and 46% for the six different laboratories in the two different runs (Table 2). For the different populations, 87% of the samples from the high-prevalence area and 0.4% of the samples from the low-prevalence area were seropositive using this test (Table 3).

**Table 2:** The percentage of seropositive serum samples (*n* = 180) for *M. bovis* infections in two duplicate runs of the ID Screen® ELISA (using a cut-off for the S/P coefficient ≥ 60% as suggested by the manufacturer together with the kit) and the BIO K302 ELISA (using a cut-off for the S/P coefficient > 37% as suggested by the manufacturer together with the kit) at the six laboratories participating in the ring trial

	*ID Screen® ELISA*			*BIO K302 ELISA*		
Lab no.	Duplicate 1 (%)	Duplicate 2 (%)	Mean of the two duplicates (%)	Duplicate 1 (%)	Duplicate 2 (%)	Mean of the two duplicates (%)
1	43.3	43.3	43.3	20.6	20.6	20.6
2	45.0	44.4	45.0	54.4	65.6	61.1
3	42.2	43.3	42.8	38.9	26.7	32.8
4	46.1	45.0	45.6	23.9	23.3	23.3
5	43.3	43.3	43.3	16.1	16.1	16.7
6	42.8	42.2	42.8	19.4	20.0	20.0
All	43.8	43.6	43.8	28.9	28.7	29.1

**Table 3:** Results of the ID Screen® ELISA and the BIO K302 ELISA as performed by the six laboratories each testing the same 180 serum samples. The mean of the two duplicate runs was used to categorise the sample as seropositive or seronegative using a cut-off for the S/P coefficient suggested by the manufacturer together with the kit (≥ 60% for the ID Screen® ELISA, and > 37% for the BIO K302 ELISA). Serum samples originated from cattle populations where *M. bovis* is known to be prevalent (the high-prevalence area: Finland, France, the Netherlands and the United Kingdom) and from a cattle population which was considered highly unlikely to have been exposed to *M. bovis* (the low-prevalence area: northern Sweden)

	Number (%) of ELISA tests					
	*ID Screen® ELISA*			*BIO K302 ELISA*		
ELISA result	High prevalence area	Low prevalence area	All samples	High prevalence area	Low prevalence area	All samples
Positive	471 (87.2)	2 (0.4)	473 (43.8)	248 (45.9)	64 (11.9)	312 (28.8)
Negative	69 (12.8)	538 (99.6)	607 (56.2)	292 (54.1)	476 (88.1)	768 (71.1)
All	540	540	1080	540	540	1080

#### ELISA II: the BIO K302 ELISA

With the BIO K302 ELISA, 29% of the samples had an S/P % > 37% and were categorised as seropositive using the cut-off recommended by the manufacturer. The proportion of positive test results varied between 16 and 66% for the six different laboratories in the two different runs (Table 2). Looking at the different populations, 46% of the samples from the high-prevalence area and 12% of the samples from the low-prevalence area were seropositive using the BIO K302 ELISA (Table 3).

### Statistical analysis

#### Precision of the two ELISA tests

For the ID Screen® ELISA, the categorisation between the two duplicate runs for each serum sample at each laboratory was generally preserved. However, on eight occasions, affecting six of the individual serum samples analysed (*n* = 180) and five of the laboratories, the categorisation differed between the first and the second test run. Data are provided in a table in Additional file 1.

For the BIO K302 ELISA, the categorisation was much less preserved between the two duplicate runs and on 64 occasions (affecting 56 out of the 180 serum samples, and all laboratories) the categorisation differed between the first and second run. Data are provided in a table in Additional file 1.

#### Accuracy of the three diagnostic tests

The accuracy of WB and the two ELISA kits were estimated by LCA using both informative priors as well as uniform distribution and estimating co-variances of the three diagnostic tests. All LCA models converged according to the diagnostic plots. The difference between the model using informative priors and the model using uniform distribution was minor but both models are presented in Table 4 for comparison. In general, the co-variances between the three tests for both *Se* and *Sp* were negligible (≤0.5%) apart from between WB and the ID Screen® ELISA (cov_Se_ = 5.4%; model with informative priors). Therefore, only the model assuming co-variance between WB and the ID Screen® ELISA is presented in Table 4. The *Se* of WB, the ID Screen® ELISA and the BIO K302 ELISA was estimated to be 91.8, 93.5 and 49.1%, respectively. *Sp* was 99.6, 98.6 and 89.6% respectively, based on the model using informative priors. With regards to the non-overlapping posterior credibility intervals (PCI), both WB and the ID Screen® ELISA had significantly higher *Se* and *Sp* than the BIO K302 ELISA (Table 4).

**Table 4:** Posterior median and 95% posterior credibility interval (95% PCI) of sensitivity and specificity for western blot analysis (WB), the ID Screen® ELISA and the BIO K302 ELISA obtained from latent class analysis assuming conditional independence between tests and using informative or uniform priors

	Informative priors		Uniform priors	
	Median	95% PCI	Median	95% PCI
Sensitivity & specificity				
Sensitivity WB	0.918	[0.879; 0.950]	0.935	[0.892; 0.973]
Specificity WB	0.996	[0.987; 1.00]	0.999	[0.993; 1.00]
Sensitivity ID Screen®	0.935	[0.898; 0.965]	0.952	[0.910; 0.990]
Specificity ID Screen®	0.986	[0.976; 0.994]	0.994	[0.985; 0.999]
Sensitivity BIO K302	0.491	[0.447; 0.535]	0.493	[0.448; 0.538]
Specificity BIO K302	0.896	[0.872; 0.918]	0.879	[0.849; 0.905]
Covariances				
Cov_Se(WB*IDScreen®)_	0.054	[0.024; 0.072]	0.038	[0.005; 0.074]
Cov_Sp(WB*IDScreen®)_	0.008	[0.000; 0.018]	0.000	[0.000; 0.004]

## Discussion

With few commercial *M. bovis* ELISAs available, several studies have applied in-house ELISAs to detect *M. bovis* antibodies in serum and milk [12, 14, 16, 18, 23, 24]. Initially, we used our set of sera to evaluate an in-house indirect ELISA using the MilA protein as antigen [17]. High intra-laboratory variation did not permit meaningful comparisons between the six participating laboratories (data not shown). For similar method transfer concerns, an in-house ELISA using a whole cell antigen preparation which has been in use for several years by one of the participating laboratories [13] was also not included for inter-laboratory comparison in this study. Instead, two available commercial ELISA systems, one of which has become widely used internationally, were chosen. A whole cell WB was used as a third diagnostic method to allow latent class statistical analysis as the information required to clinically and microbiologically confirm *M. bovis* infection was not available for all the serum samples included in the study.

To our knowledge, this is the first study to present an inter-laboratory comparison of the BIO K302 ELISA (BioX Diagnostics) and the ID Screen® ELISA (IDVet) for serological detection of *M. bovis* infections in cattle. Overall, the results demonstrated high *Se* and high *Sp* for the ID Screen® ELISA, while the same values for the BIO K302 ELISA were significantly lower, based on non-overlapping 95% PCIs (Table 4). These findings are broadly in agreement with previous studies which report a lower *Se* for the BIO K302 ELISA compared with other ELISAs [12, 17, 21]. In addition, Schibrowski et al. (2018) reported a similar *Se* (47%) for the BIO K302 ELISA while using the manufacturer’s recommended cut-off and analysing serum samples from exposed and non-exposed cattle from three different countries [10, 11].

The difference in diagnostic accuracy of the two ELISAs was reflected in the percentage of seropositive cattle from the high-prevalence area where there was a difference in proportion of positive tests obtained, varying from 87.2 to 45.9%, depending on whether the ID Screen® ELISA or the BIO K302 ELISA was used. By comparison, WB identified 83.5% of the samples from the high-prevalence area as seropositive for *M. bovis*. The difference in accuracy was also observed for the categorisation of the serum samples from the low-prevalence area. Only WB categorised all samples from the low-prevalence area as seronegative for *M. bovis*. For the ID Screen® ELISA, 0.4% of the serum samples from northern Sweden were categorised as positive for *M. bovis*, but for the BIO K302 ELISA the corresponding percentage was substantially higher at 12%. These samples were included in the study since they originate from a population of cattle highly unlikely to ever have been exposed to *M. bovis* [25].

It has previously been suggested that the manufacturer’s recommended cut-off of 37% for the BIO K302 ELISA samples is too low, and a cut-off of 50% would reduce the false positive results [21]. However, the study in question was based on bulk tank milk samples and cannot be directly extrapolated to serum diagnostics. Further, in our study, for the set of sera from the high-prevalence area, the percentage of seropositive samples as well as the *Se* value obtained were both markedly lower for the BIO K302 ELISA than WB and ID Screen® ELISA. Raising the cut-off to 50% would result in even lower *Se* for the BIO K302 ELISA and such changes should not be attempted without prior optimisation of the ELISA kit.

To assess the precision of the three tests and allow for better comparison between the six laboratories, duplicate runs were performed for both the ELISA tests. For the ID Screen®, results between the duplicate runs were very similar, with only six samples differing in categorisation at one or two laboratories. For the BIO K302, on the other hand, 31% of the samples differed in categorisation between the two runs, especially at two laboratories (these data were not specifically centred around the cut-off nor pair-wise close to each other; see data in Additional file 1). Use of the same batch of each ELISA kit by all participating laboratories for the study was designed to negate effects of batch variability for each of the ELISAs. Moreover, technical issues that may have contributed to the observed differences for the repeated testing of samples might reasonably have been expected to affect both tests similarly, which was not the case here. Unfortunately, the study design did not enable further evaluation of this unexpected variation, specifically whether the source was associated with the robustness of the assay per se or at the local laboratory level.

One of the ELISA systems included in this study, BIO K302, has previously been evaluated on BTM samples [21, 26, 27] as well as on serum samples [17, 27]. BTM samples can, if the ELISA is sensitive and specific, be an excellent tool for herd diagnosis of several systemic infections in dairy herds [28–30]. However, previous studies have found that this is more challenging for *M. bovis*; while cattle suffering from *M. bovis* mastitis have been found to have high antibody titres both in milk and serum Moreover, the serological status of young stock may not be well reflected by testing of BTM samples, and milk antibody measurements only have diagnostic utility for cows with mastitis and are unsuitable for other clinical manifestations of *M. bovis* infections [31]. Information demonstrating correlation in both matrices is still lacking and warrants further study. Thus, in herds where no clinical mastitis or no lactating cows are present, ELISA analysis must rely on serum sampling instead of milk samples. For this reason, it is crucial that the chosen ELISA method has been validated for detection of antibodies in serum with high accuracy and high precision. Since the ID Screen® ELISA showed a higher precision and accuracy than the BIO K302 ELISA in this study, the ID Screen® ELISA seems promising as a test for reliable determination of infection status for control programs, disease management and research purposes in the future. However, further evaluation of the ID Screen® ELISA under field conditions, on other sample types, such as milk in animals of known disease status, and comparative microbiological testing is warranted. In addition, a longitudinal study similar to that described by Petersen et al., [31], evaluating the antibody response between individual animals, as well as paired samples from the same individual, would be of great value in the future.

Despite reported failures of antimicrobial chemotherapy to control *M. bovis* infection, there are currently no effective vaccines available against *M. bovis* in Europe and the reported performance of the vaccines currently licensed in the USA is rather poor [1, 32]. Efforts are ongoing to identify antigens that will induce a protective immunity. Antigenic proteins conserved across different strains are most likely to be targeted as potential *M. bovis* vaccine candidates and hence are often similar to proteins of greatest interest as targets for immunodiagnostic tools. A future challenge facing producers of serological tests for *M. bovis* will be to ensure accurate detection of serological response to ensure effective discrimination between vaccinated and naturally infected cattle. It also highlights that despite limited new developments in *M. bovis* serological testing, regular assessment of the performance of available serological diagnostic tools is required to take into account minor changes to test components and the diversity and evolution of strains causing infection worldwide. Future developments should also consider the potential existence of different vaccination strategies.

## Conclusions

With increased awareness of the importance of *M. bovis* in bovine respiratory disease and mastitis, there is growing requirement for readily available, reproducible serological assays offering high precision and accuracy for diagnosis of *M. bovis* infection in cattle herds globally. Differences in the performance of the two commercially available ELISAs demonstrates that limitations exist with the use of such tests and highlights the importance undertaking regular assessment of performance, even when commercial tests are being used. Using a test with a high *Se* and *Sp* proven by inter-laboratory comparison, such as the ID Screen® ELISA, can provide improved knowledge on the prevalence of *M. bovis* in different cattle populations. Through a combination of validated diagnostic tools, well assessed sample strategies and information on clinical symptoms on herd and animal level, we can improve monitoring of *M. bovis* in cattle herds and enhance our understanding of the epidemiology of *M. bovis* infections.

## Methods

### Participating institutes

Six Animal Health Institute laboratories from six different European countries participated in the *M. bovis* ELISA ring trial: the Animal and Plant Health Agency (APHA), UK; the National Veterinary Institute, Technical University of Denmark (DTU), Denmark; the Finnish Food Safety Authority (Evira), Finland; the French Agency for Food, Environmental and Occupational Health & Safety (ANSES), France; the National Veterinary Institute (SVA), Sweden and the Wageningen Bioveterinary Research (WBVR), The Netherlands. The laboratories were anonymously allocated a number 1 to 6.

### Origin and distribution of serum samples

For evaluation of the diagnostic tests, a serum panel comprising 180 cattle serum samples was collated. Half of the panel (*n* = 90) comprised sera derived from animals from *M. bovis* infected farms in France, Finland, the UK and the Netherlands where *M. bovis* is known to be prevalent [6, 33–35]. This area will be hereafter called the “high-prevalence area” (Table 5). The farms from which the samples were drawn were located in different geographical areas within each country and, where information was available, originated from cattle with different disease manifestations including respiratory disease, mastitis and arthritis. Most, but not all, of these sera had previously been determined as positive for *M. bovis* by the tests commonly used by the respective laboratories and from clinical information from the herd. Additionally, where available PCR data confirming the presence of the organism by PCR was used to inform selection. A small number of samples from the high-prevalence area that had previously tested negative, or were only weakly positive but taken from herds that included animals with moderate-to-high titres, were also included. The remaining 90 serum samples represented a cattle population from northern Sweden, where *M. bovis* has never been diagnosed, and is therefore considered highly unlikely to have been exposed to *M. bovis* infection [25]. This area will hereafter be known as the “low-prevalence area*”* (Table 5).

**Table 5:** Country of origin, number of samples, clinical disease manifestation, age group of cattle sampled, number of farms sampled, year of collection as well as prevalence area categorisation based on anticipated *M. bovis* seroprevalence for the 180 serum samples used in this study

Country	Number of samples	Clinical signs	Age	Number of farms	Year(s) of collection	Prevalence area
Finland	30	Mastitis^b^	Various	3	2015 to 2016	High
France	22^a^	Pneumonia	Veal calves	9	2014	High
UK	28	Various	Various	18	2013 to 2017	High
The Netherlands	10	Pneumonia	Veal calves	10	2014	High
Sweden	90^a^	None	NA	NA	2013	Low
Total no samples	180					

^a^Pooled sera, two individual sera per pool

^b^Samples from infected animals from farms with few mastitis cases

*NA* data not available

All sera were sent to laboratory 4 where they were randomised, aliquoted into individual tubes, labelled with numbers 1 to 180 and sent to each participating laboratory for blinded testing (including laboratory 4). The samples were dispatched on dry ice and each laboratory stored the serum samples at, or below, − 20 °C until analysis. Laboratory 3 received a supplemental volume of each serum sample for WB.

### Three serological methods

#### Western blot analysis

*M. bovis* strain L15762, belonging to the main subtype currently circulating in France [33, 34], was cultivated at 37 °C for 48 h in modified PPLO broth [36]. Cells were harvested by centrifugation at 12,000 x g for 20 min at 4 °C, washed three times in phosphate buffered saline (PBS) and protein content estimated using the Pierce BCA protein assay kit (ThermoFisher Scientific, Illkirch, France). Twenty μg of protein, suspended in Laemmli buffer, was loaded per well on a miniprotean TGX 10% gel (BioRad, Marnes-La-Coquette, France) for SDS-PAGE analysis. Electrophoresis was performed at 125 V until the bromophenol blue reached the bottom of the gel. Subsequent blotting and immuno-detection steps were conducted as previously described [37]. For the positive control, serum from a veal calf with respiratory disease, determined to be positive for *M. bovis* following real-time PCR (MPBO50 kit from ThermoFisher Scientific) and cultural examination of nasal swabs and BALF, sampled at day 21 after clinical onset, was used. The negative control originated from a pool of sera from two veal calves taken prior to introduction to a feedlot (day 0), when calves were of approximately 15 days old. Both animals tested negative by an *M. bovis*-specific PCR [38], with the herd confirmed seronegative (ELISA BIO K302) at day 0 and again 40 days later). A serum sample was considered positive if its WB profile contained two discrete immunogenic bands at 50 and 85 kDa (Fig. 1).

#### ELISA I: ID screen® ELISA

Serum samples were analysed with the ID Screen® *Mycoplasma bovis* indirect ELISA kit according to the instructions of the manufacturer (IDvet, Grabels, France). All laboratories used the same manufacturers’ batch of ELISA kit and all reagents, including positive and negative controls, were provided by the manufacturer as a part of the kit. The following procedures were followed by all participating laboratories. The serum samples were thawed and diluted 1:40 in dilution buffer in the pre-coated plates. Positive and negative controls were added in duplicate to each plate. After incubation for 45 min at room temperature (RT, recommended by the manufacturer to be 21 +/− 5 °C), each well was washed three times with wash solution prior to addition of 100 μL anti-bovine horseradish peroxidase (HRP) conjugate. After 30 min incubation at RT, the plates were again washed three times before 100 μL 3,3′,5,5′-tetramethylbenzidine (TMB) substrate solution was added to each well. The plates were incubated 15 min in the dark at RT before the reaction was stopped by adding 100 μL stop solution. The optical density (OD) was measured at 450 nm. The test was considered valid if the mean value of the positive control was greater than 0.350, and the ratio between the mean positive control and the mean negative control was greater than three. All serum samples were run in duplicate on separate plates and for each serum sample in each run the sample-to-positive percentage (S/P %) was calculated using the formula:

$$ \mathrm{S}/\mathrm{P}\%=\left(\left({\mathrm{OD}}_{sample}-{\mathrm{OD}}_{mean\ negative\ control}\right)/\left({\mathrm{OD}}_{mean\ positive\ control}-{\mathrm{OD}}_{mean\ negative\ control}\right)\right)\ \mathrm{x}\ 100 $$

The S/P % for each sample for each run was used to categorise the sample as positive or negative using the cut-off value provided by the manufacturer (positive if the S/P % ≥ 60%).

#### ELISA II: the BIO K302 ELISA

Serum samples were analysed with the *Mycoplasma bovis* BIO K302 according to manufacturers’ instructions (Bio-X Diagnostics, Rochefort, Belgium). All laboratories used the same batch of ELISA kit and all reagents, including positive and negative controls, were provided as part of the kit. The following procedure was used. The serum samples were thawed and diluted 1:100 so that 100 μL of each diluted serum sample was added in duplicate to the pre-coated plates. Positive and negative controls were added in duplicate to each plate. After incubation for 1 h at RT, the plates were washed three times with wash solution and 100 μL of conjugate solution (HRP conjugated Protein G) diluted 1:50 was added to each well. The plates were again incubated for 1 h at RT (recommended by the manufacturer to be 21 +/− 3 °C) before washing three times followed by addition of 100 μL of TMB reagent solution to each well. The plates were incubated 10 min at RT in the dark before the reaction was stopped with 50 μL of stop solution (1 M phosphoric acid). The OD was measured at 450 nm. The test was considered valid if the difference between the mean positive and the mean negative control was greater than 0.7 and the mean negative serum had an OD_450_ of less than 0.4. All serum samples were run in duplicate on separate plates and for each serum sample in each run an S/P % was calculated as described above for the ID Screen® ELISA according to manufacturers’ instructions. The S/P % for each sample for each run was again used to categorise the sample as positive or negative using the cut-off value provided by the manufacturer (positive if S/P % > 37%).

### Reporting of results

Each of the six participating laboratories performed the two commercially available ELISAs, but only one laboratory (laboratory 3) performed the WB. A standardised result recording template was completed by each of the laboratories. Data was collated and statistically analysed using dedicated expertise at one of the participating laboratories.

### Statistical analysis

#### Precision

The ability of the two ELISA systems to consistently categorise the sample as positive or negative was evaluated by comparing the categorisation in each of the two duplicate runs for each sample at each of the six participating laboratories.

#### Accuracy

For each serum sample and ELISA, the mean S/P % from the two duplicate runs were used to categorise the sample as positive and negative for each laboratory, again using the cut-off value provided by the two manufacturers. The results from the six different laboratories were then used to evaluate the diagnostic performance of each of the three tests for serodiagnosis of *M. bovis*. In the absence of a gold-standard reference test for *M. bovis* antibodies in cattle, a LCA using a Bayesian formulation was selected as the preferred method to estimate the diagnostic sensitivities (*Se*) and specificities (*Sp*) of the three diagnostic tests [39–42]. The serum samples were separated into two subpopulations based on their origin from the afore-mentioned high-prevalence or the low-prevalence areas (Table 5).

The LCA was performed in OpenBUGS 3.2.2 rev 1063 (OpenBUGS, 2010–2011 Members of Open BUGS Project Management Group) using a Markov Chain Monte Carlo (MCMC) sampling algorithm (Gibbs sampling algorithm) to obtain a random sample from the joint posterior distribution of all model parameters. Informative prior beta distributions for *Se* and *Sp* were estimated based on previous *M. bovis* serological and bulk tank milk (BTM) studies [10, 12, 17, 21], and the alpha and beta parameters for the beta distributions were estimated using the calculator on the EpiTools website [43]. The input values for the prior distributions are summarised in Table 6. In addition to the model with informative priors, a model with uniform distribution for all parameters was also applied (β (1,1).

**Table 6:** Values used as priors in the latent class analysis for sensitivity (Se) and specificity (Sp) of the western blot analysis (WB), the ID Screen® ELISA and the BIO K302 ELISA, respectively, together with the estimated alpha and beta parameters (α; β) for the beta distribution of these priors

Diagnostic test	Se	β (α; β)	Sp	β (α; β)
WB	0.72 (o.16–0.96)	1.8413; 1.3272	0.90 (0.56–1.00)	6.895; 1.655
ID Screen®	0.90 (0.80–1.00)	42.5732; 5.6192	0.95 (0.90–1.00)	99.6983; 6.1946
BIO K302	0.48 (0.30–0,65)	11.1876; 12.0365	0.96 (0.90–1.00)	128.4285; 6.3095

In the LCA, three MCMC chains with different initial values were compiled for 50,000 iterations of the model, discarding the first 15,000 iterations as the burn-in phase. A thinning of 1 in 10 was applied. The time-series plots of the variables, the Gelman-Rubin diagnostic plots and the autocorrelation plots were all visually inspected for model evaluation. As estimation of *Se* and *Sp*, the covariance between the three diagnostic tests, as well as the seroprevalence in each subpopulation of samples, the median of the posterior distribution was applied. As estimates of the 95% posterior credibility intervals for each variable, the 2.5 and 97.5 points were used.

## Supplementary information

**Additional file 1.** Table with the sample number, the S/P % for the two duplicate runs (duplicate 1 and 2, respectively) as well as the categorisation (cat.) based on the cut-off suggested by the manufacturer for the in total 72 duplicates where the categorisation was not preserved between the two runs.

## Data Availability

All datasets supporting our findings are available from the corresponding author on reasonable request.

## Abbreviations

BTM: Bulk tank milk
ELISA: Enzyme-linked immunosorbent assay
LCA: *Latent class analysis*
MCMC: Markov Chain Monte Carlo
OD: Optical density
PBS: Phosphate buffered saline
PCI: Posterior credibility intervals
PCR: Polymerase chain reaction
RT: Room temperature
S/P %: Sample-to-positive percentage
*Se*: Sensitivity
*Sp*: Specificity
TMB: Tetramethylbenzidine
WB: Western blot analysis
