# Supplementary material for: A European inter-laboratory trial to evaluate the performance of three serological methods for diagnosis of Mycoplasma bovis infection in cattle using latent class analysis
Source: BMC Vet Res. 2019 Oct 25;15:369. doi: 10.1186/s12917-019-2117-0 (PMC6814985; doi:10.1186/s12917-019-2117-0)
Supplement: Supplementary file 1 — Additional file 1. Table with the sample number, the S/P % for the two duplicate runs (duplicate 1 and 2, respectively) as well as the categorisation (cat.) based on the cut-off suggested by the manufacturer for the in total 72 duplicates where the categorisation was not preserved between the two runs. [file 12917_2019_2117_MOESM1_ESM.docx]

*Supplementary Table: The sample number, the S/P % for the two duplicate runs (duplicate 1 and 2, respectively) as well as the categorisation (cat.) based on the cut-off suggested by the manufacturer for the in total 72 duplicates where the categorisation was not preserved between the two runs.*

|  | **BIO K302** | | | |  | **ID Screen** | | | |
| --- | --- | --- | --- | --- | --- | --- | --- | --- | --- |
| **Sample** | *duplicate 1* | | *duplicate 2* | |  | *duplicate 1* | | *duplicate 2* | |
|  | S/P % | Cat. | S/P % | Cat. |  | S/P % | Cat. | S/P % | Cat. |
| 2 | 36 | neg | 43 | pos |  |  |  |  |  |
| 3 | 43 | pos | 36 | neg |  |  |  |  |  |
| 4 | 39 | pos | 29 | neg |  |  |  |  |  |
| 5 | 34 | neg | 46 | pos |  |  |  |  |  |
| 7 | 47 | pos | 26 | neg |  |  |  |  |  |
| 18 |  |  |  |  |  | 64 | pos | 50 | neg |
| 20 | 54 | pos | 36 | neg |  |  |  |  |  |
| 33 | 33 | neg | 46 | pos |  |  |  |  |  |
| 38 | 30 | neg | 38 | pos |  |  |  |  |  |
| 41 | 29 | neg | 42 | pos |  |  |  |  |  |
| 49 | 50 | pos | 32 | neg |  |  |  |  |  |
| 55 | 29 | neg | 38 | pos |  |  |  |  |  |
| 63 | 41 | pos | 32 | neg |  |  |  |  |  |
| 78 |  |  |  |  |  | 75 | pos | 59 | neg |
| 79 | 39 | pos | 28 | neg |  |  |  |  |  |
| 82 | 41 | pos | 36 | neg |  |  |  |  |  |
| 87 | 33 | neg | 38 | pos |  |  |  |  |  |
| 91 | 49 | pos | 19 | neg |  |  |  |  |  |
| 92 | 28 | neg | 53 | pos |  |  |  |  |  |
| 97 | 34 | neg | 57 | pos |  |  |  |  |  |
| 97 | 86 | pos | 33 | neg |  |  |  |  |  |
| 101 | 18 | neg | 43 | pos |  |  |  |  |  |
| 103 | 19 | neg | 51 | pos |  |  |  |  |  |
| 104 | 30 | neg | 89 | pos |  | 59 | neg | 65 | pos |
|  |  |  |  |  |  | 62 | pos | 45 | neg |
| 105 | 17 | neg | 59 | pos |  |  |  |  |  |
| 105 | 43 | pos | 24 | neg |  |  |  |  |  |
| 113 | 21 | neg | 56 | pos |  | 54 | neg | 84 | pos |
|  | 53 | pos | 34 | neg |  | 55 | neg | 61 | pos |
| 118 | 32 | neg | 47 | pos |  | 145 | pos | 36 | neg |
| 119 |  |  |  |  |  | 66 | pos | 59 | neg |
| 122 | 47 | pos | 25 | neg |  |  |  |  |  |
| 122 | 29 | neg | 40 | pos |  |  |  |  |  |
| 126 | 33 | neg | 42 | pos |  |  |  |  |  |
|  | 38 | pos | 20 | neg |  |  |  |  |  |
| 129 | 21 | neg | 40 | pos |  |  |  |  |  |
|  | 50 | pos | 28 | neg |  |  |  |  |  |
| 132 | 40 | pos | 34 | neg |  |  |  |  |  |
| 135 | 51 | pos | 35 | neg |  |  |  |  |  |
| 139 | 64 | pos | 22 | neg |  |  |  |  |  |
|  | 30 | neg | 71 | pos |  |  |  |  |  |
| 142 | 51 | pos | 29 | neg |  |  |  |  |  |
| 143 | 66 | pos | 35 | neg |  |  |  |  |  |
| 144 | 52 | pos | 20 | neg |  |  |  |  |  |
| 145 | 64 | pos | 30 | neg |  |  |  |  |  |
| 147 | 46 | pos | 27 | neg |  |  |  |  |  |
| 154 | 16 | neg | 38 | pos |  |  |  |  |  |
| 157 | 28 | neg | 48 | pos |  |  |  |  |  |
| 159 | 47 | pos | 32 | neg |  |  |  |  |  |
|  | 74 | pos | 33 | neg |  |  |  |  |  |
| 162 | 23 | neg | 52 | pos |  |  |  |  |  |
|  | 45 | pos | 28 | neg |  |  |  |  |  |
| 163 | 33 | neg | 39 | pos |  |  |  |  |  |
| 165 | 40 | pos | 22 | neg |  |  |  |  |  |
| 166 | 50 | pos | 25 | neg |  |  |  |  |  |
| 167 | 35 | neg | 39 | pos |  |  |  |  |  |
| 168 | 46 | pos | 27 | neg |  |  |  |  |  |
| 169 | 48 | pos | 20 | neg |  |  |  |  |  |
| 170 | 53 | pos | 34 | neg |  |  |  |  |  |
| 171 | 37 | pos | 21 | neg |  |  |  |  |  |
|  | 12 | neg | 73 | pos |  |  |  |  |  |
| 172 | 7 | neg | 43 | pos |  |  |  |  |  |
| 174 | 39 | pos | 21 | neg |  |  |  |  |  |
|  | 11 | neg | 76 | pos |  |  |  |  |  |
| 175 | 7 | neg | 46 | pos |  |  |  |  |  |
| 176 | 23 | neg | 93 | pos |  |  |  |  |  |
| 179 | 32 | neg | 67 | pos |  |  |  |  |  |
| 180 | 20 | neg | 38 | pos |  |  |  |  |  |
